# Using the Kirkpatrick Model to Evaluate the Effect of a Primary Trauma Care Course on Health Care Workers’ Knowledge, Attitude, and Practice in Two Vietnamese Local Hospitals: Prospective Intervention Study

**DOI:** 10.2196/47127

**Published:** 2024-07-23

**Authors:** Ba Tuan Nguyen, Van Anh Nguyen, Christopher Leigh Blizzard, Andrew Palmer, Huu Tu Nguyen, Thang Cong Quyet, Viet Tran, Marcus Skinner, Haydn Perndt, Mark R Nelson

**Affiliations:** 1 Menzies Institute for Medical Research, University of Tasmania, Hobart, Australia; 2 Department of Medical Education and Skills Laboratory, Hanoi Medical University, Hanoi, Vietnam; 3 Department of Anaesthesia, Hanoi Medical University, Hanoi, Vietnam; 4 Tasmanian School of Medicine, University of Tasmania, Hobart, Australia; 5 Emergency Department, Royal Hobart Hospital, Tasmanian Health Service, Hobart, Australia; 6 Department of Anaesthesia, Royal Hobart Hospital, Tasmanian Health Service, Hobart, Australia

**Keywords:** trauma care, emergency medicine, primary trauma care course, short course, medical education, trauma, emergency, urgent, professional development, workshop, injury, injured, injuries, primary care

## Abstract

**Background:**

The Primary Trauma Care (PTC) course was originally developed to instruct health care workers in the management of patients with severe injuries in low- and middle-income countries (LMICs) with limited medical resources. PTC has now been taught for more than 25 years. Many studies have demonstrated that the 2-day PTC workshop is useful and informative to frontline health staff and has helped improve knowledge and confidence in trauma management; however, there is little evidence of the effect of the course on changes in clinical practice. The Kirkpatrick model (KM) and the knowledge, attitude, and practice (KAP) model are effective methods to evaluate this question.

**Objective:**

The aim of this study was to investigate how the 2-day PTC course impacts the satisfaction, knowledge, and skills of health care workers in 2 Vietnamese hospitals using a conceptual framework incorporating the KAP model and the 4-level KM as evaluation tools.

**Methods:**

The PTC course was delivered over 2 days in the emergency departments (EDs) of Thanh Hoa and Ninh Binh hospitals in February and March 2022, respectively. This study followed a prospective pre- and postintervention design. We used validated instruments to assess the participants’ satisfaction, knowledge, and skills before, immediately after, and 6 months after course delivery. The Fisher exact test and the Wilcoxon matched-pairs signed rank test were used to compare the percentages and mean scores at the pretest, posttest, and 6-month postcourse follow-up time points among course participants.

**Results:**

A total of 80 health care staff members attended the 2-day PTC course and nearly 100% of the participants were satisfied with the course. At level 2 of the KM (knowledge), the scores on multiple-choice questions and the confidence matrix improved significantly from 60% to 77% and from 59% to 71%, respectively (*P*<.001), and these improvements were seen in both subgroups (nurses and doctors). The focus of level 3 was on practice, demonstrating a significant incremental change, with scenarios checklist points increasing from a mean of 5.9 (SD 1.9) to 9.0 (SD 0.9) and bedside clinical checklist points increasing from a mean of 5 (SD 1.5) to 8.3 (SD 0.8) (both *P*<.001). At the 6-month follow-up, the scores for multiple-choice questions, the confidence matrix, and scenarios checklist all remained unchanged, except for the multiple-choice question score in the nurse subgroup (*P*=.005).

**Conclusions:**

The PTC course undertaken in 2 local hospitals in Vietnam was successful in demonstrating improvements at 3 levels of the KM for ED health care staff. The improvements in the confidence matrix and scenarios checklist were maintained for at least 6 months after the course. PTC courses should be effective in providing and sustaining improvement in knowledge and trauma care practice in other LMICs such as Vietnam.

## Introduction

### Health Care Burden of Road Trauma

Road traffic trauma is a leading cause of morbidity and mortality globally [1]. Road trauma is responsible for 1.3 million deaths and 20-50 million injuries annually with 90% of these occurring in low- and middle-income countries (LMICs) [2,3]. It is predicted that the prevalence of road trauma will increase to become the third leading cause of death by 2030 in LMICs [4]. While there are many contributing factors to the higher impact of road trauma in LMICs than in high-income countries, including infrastructure, vehicle design, underdevelopment of health care systems, and lack of trauma care education [5], the latter factor was highlighted among the 5 key World Health Organization targets for the first decade of action on road safety for LMICs from 2011 to 2020 [6,7]. If this target is achieved, it is estimated that one-third of annual global trauma deaths could be prevented [8,9].

As in other LMICs, road traffic trauma is a major public health problem in Vietnam [10]. In the last 15 years, approximately 10 people per 100,000 population have been killed in road accidents in Vietnam each year, with an equal number hospitalized [11,12]. Road traffic injury is the second most common cause of death for people in Vietnam in the age group of 5‐14 years, representing the most vulnerable and dependent population, and is the most common cause of death and disability for those in the age group of 15‐49 years, representing the most productive population [13]. The Vietnamese health care system is built on a “pine tree” model in which a trauma center has responsibility for various “satellite” hospitals and receives patients experiencing severe trauma. In a satellite or frontline hospital, the staff first encountering a trauma patient may be a surgeon, nurse, anesthetist, or general practitioner who may not be trained in a trauma subspecialty. Indeed, the trauma training system in Vietnam is not adapted to this circumstance [14]. There is therefore a need for a training system to solve this education gap.

There are several trauma training courses that have been delivered around the world, such as the Advanced Trauma Life Support (ATLS), Trauma Team Training, and Primary Trauma Care (PTC) courses. Several trauma courses are currently being used in LMICs effectively, resulting in increases in knowledge and at a lower reported cost than the gold-standard ATLS course [15]. Among these, the PTC course is designed and structured to fit within health care systems in LMICs such as Vietnam. In particular, the PTC course requires minimal resources and is therefore sustainable for these countries [16]. For this reason, since 2007, the PTC course has been run in many regional and provincial areas of Vietnam, including Binh Dinh (2007), Hanoi (2008), Ninh Binh (2008), and Ho Chi Minh City (2018) [17-19]. However, none of these Vietnamese courses has been evaluated with respect to the effect on the clinical practice of health care staff.

### Education Frameworks

One of the methods commonly used to assess educational programs is the 4-level Kirkpatrick model (KM). This model was developed by Kirkpatrick in 1959 and has since been widely used to evaluate the effectiveness of continuing education in many fields, including medicine [15]. The KM evaluates the training outcomes of a course at 4 levels depending on the amount of time the evaluation is undertaken after the course. Level 1 evaluates trainees’ satisfaction toward the instructors and the training program, level 2 assesses trainees’ learning of professional knowledge or skills, level 3 measures the changes in trainees’ behavior or performance, and level 4 quantifies the improvement of the outcomes closely linked to the training program that will work effectively in the long term. This model has been considered a suitable assessment tool for educational programs as it has a simple process, measures a limited number of variables, and does not depend on individual variables [20].

Moreover, previous studies in education have identified that trainees’ knowledge of issues and possession of skills are required for this knowledge to transfer into behavioral change [21], and that positive attitudes and behaviors could lead an individual to be better motivated toward an issue [22]. Since trauma care is a common and vital practice of many health care staff working at the frontline of the Vietnamese health care system, the effectiveness of PTC training courses should be improved and consolidated through knowledge, attitude, and practice (KAP)–based education. As such, the KAP model can be an appropriate approach to help identify knowledge gaps, attitude barriers, and practice patterns that may facilitate understanding the knowledge and practice of health care professionals after attending a PTC training course [23].

**Figure 1.**
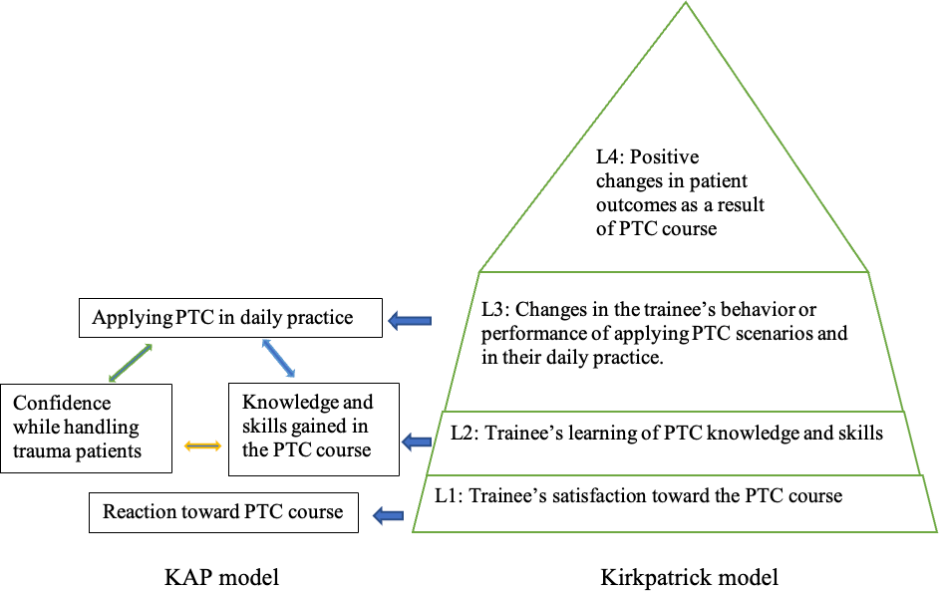
Theoretical framework of the study based on the KAP and Kirkpatrick models of evaluation. KAP: knowledge, attitude, and practice; PTC: Primary Trauma Care; L1: level 1; L2: level 2; L3: level 3; L4: level 4.

Previous studies have used the KM and KAP model as theoretical frameworks to evaluate the effectiveness of training courses in the health care field [20,24]. In applying these models for this study, we aimed to: (1) measure the outcomes of the PTC course by asking the participants to clarify their reaction to the relevance and usefulness of the course (level 1 of the KM); (2) test the knowledge and level of confidence at the precourse, immediately postcourse, and 6-month postcourse time points (level 2 of the KM); and (3) observe changes in the practice of participants in scenarios and handling trauma patients in their daily work (level 3 of the KM) (Figure 1). Level-4 changes have not yet been assessed but are the subject of an ongoing investigation.

To gain a more comprehensive understanding on the effectiveness of the education of PTC training courses, this study was performed to assess the impact of the PTC course on health care staff by using a conceptual framework involving two theories of evaluation: the KAP model and the 4-level KM . The specific study objectives were to (1) investigate the impact of the PTC training course on the satisfaction, confidence, and change of knowledge and skills of participants in two hospitals in Vietnam; and (2) evaluate the retention of the participants’ knowledge and changes in skills 6 months after the PTC training course.

## Methods

### Setting

This study was carried out in the emergency departments (EDs) of 2 provincial hospitals in Vietnam, the Thanh Hoa and Ninh Binh hospitals. There are 78 beds in both EDs with 86 staff comprising 35 doctors and 51 nurses.

### PTC Course

The 2-day PTC courses were delivered by local PTC instructors from Hanoi Medical University following the standard format of the PTC Foundation [25]. The PTC course included lectures, skills workshops, and case scenarios to cover a range of topics and practical skills. The objective of the course was to train participants to approach trauma patients in a sequential manner without missing life-threating symptoms and processes.

All health care staff at the 2 EDs were invited to participate in the course. To maintain an effective clinical workforce in the respective EDs, each site was divided into 2 classes that rotated and ran over 3 days. In Thanh Hoa Hospital, the courses ran from February 17 to February 20, 2021, whereas in Ninh Binh Hospital, the courses ran from March 3 to March 6, 2021. Each class received the same training and assessment. All material is available on the PTC website [25].

### Study Design

This study followed a pre- and postinterventional design. We used validated instruments to assess the participants’ satisfaction and confidence prior to the course and immediately after training, as well as to compare their level of trauma knowledge and skills at the precourse, postcourse, and 6-month follow-up time points. We also stratified participants into doctor and nurse groups for further investigation. The trial has been registered in the Australian New Zealand Clinical Trial Registry (ACTRN 12621000371897) [26].

### Outcome Measures

#### KM Level 1: Satisfaction

A self-completed questionnaire was developed to explore participants’ reactions to the PTC course. The questionnaire contained 5 items regarding the relevance and usefulness of the course. Trainees indicated their agreement with the corresponding statements using a 5-point Likert scale, with 1 indicating “strongly disagree” and 5 indicating“strongly agree” (Multimedia Appendix 1).

#### KM Level 2: Knowledge

Twenty multiple-choice questions were used to evaluate the complete teaching content in the course, which have also been used in previous evaluations [16]. Most of the items assessed the trainees’ knowledge domain in the knowledge and comprehension categories according to the Bloom taxonomy [27]. The multiple-choice questions focused on knowledge in trauma management, including the areas of head, thoracic, and abdominal trauma, requiring the trainees to remember key fundamental points from serial lectures in the course (Multimedia Appendix 2). The results were calculated as the percentage of correct answers. This multiple-choice question set was developed for the PTC program, translated into Vietnamese by local PTC instructors, and edited by experts and educators from the PTC Foundation.

A confidence matrix was also included with 8 questions assessing the level of confidence while dealing with various circumstances related to patients experiencing trauma. Each question was rated according to 5 levels of confidence, ranging from 1 (the lowest level of confidence) to 5 (the highest level of confidence). This result was also calculated as a percentage for analysis (Multimedia Appendix 3).

#### KM Level 3: Practice Skills

Scenario checklists, which included various clinical scenarios, were used to evaluate participants’ practice skills in a simulation. The participants at the 2 hospitals were divided into small groups of 6‐7 people. Each group included both doctors and nurses to replicate an emergency team on duty in the ED. The assessments were conducted using an Objective Structured Clinical Examination format [20,24] with 4 stations, each station lasting up to 10 minutes, with one observer who used a standardized checklist to rate the performance of the team. Each scenario checklist comprised 10 key actions. If the examined group achieved this action, they were given 1 point; otherwise, they received 0 points. We chose 4 scenarios for the evaluation and rotated all groups within these 4 scenarios to ensure that the maximum amount of skills were evaluated (Multimedia Appendix 4).

For the bedside clinical checklist, we used an observed checklist with 10 vital points that had been stressed in the course. This checklist was used by experienced clinicians who were local PTC instructors. To minimize observation stress, all participants were informed about the presence of the examiners before and after the course. The examiners were allocated cases randomly in both the pre- and postcourse phases (Multimedia Appendix 5).

### Statistical Analysis

We used Stata version 15.1 software for statistical analyses. The Fisher exact and Wilcoxon matched-pairs signed rank tests were used to compare percentages (multiple-choice questions and confidence matrix) and the mean scores (scenario and bedside clinical checklists) among the precourse, postcourse, and 6-month follow-up time points for the participants. A *P* value <.05 was considered statistically significant.

### Ethical Considerations

The University of Tasmania Human Research Ethics Committee approved this study (reference number H0023982) [28]. All participants who volunteered to take part in the study (without compensation) were required to sign the consent form (Multimedia Appendix 6). All data were deidentified and stored online in a password-protected Google drive of the research group’s account to ensure privacy and confidentiality.

## Results

### Participant Characteristics

Among the 86 health care staff in the EDs, 80 participated in the course; the 6 individuals who could not participate in the course were excluded owing to testing positive for COVID-19 at the time of course delivery. All participants completed the pre-and posttest assessments and the scenarios checklist. Only 57 (71%) of the 80 participants completed the joint evaluation of knowledge and scenarios checklist at the 6-month follow-up. The cohort consisted of 34 doctors (mean age 28.0, SD 2.5 years) and 46 nurses (mean age 32.0, SD 6.4 years). Nurses had more general medical work experience than doctors (mean 6.3, SD 5.6 years vs 2.4, SD 1.7 years). Male staff accounted for 68% of the doctors and 65% of the nurses.

### KM Level 1: Survey Responses

All participants responded to the survey, with 78 of the 80 participants (98%) indicating satisfaction with the course. Likewise, an equal number of respondents stated that “the course enhanced their knowledge” and 79 of the 80 participants (99%) stated that they would “suggest the course to others.” Furthermore, 77 of the 80 participants (96%) agreed that “the course was relevant to ED staff” (Table 1).

**Table 1.** Level 1 of the Kirkpatrick model: participant reactions to the Primary Trauma Care (PTC) course (N=80).

Question	Strongly disagree (1), n (%)	Somewhat disagree (2), n (%)	Neither agree or disagree (3), n (%)	Somewhat agree (4), n (%)	Strongly agree (5), n (%)
I was satisfied with the PTC course overall	1 (1)	1 (1)	0 (0)	8 (10)	70 (88)
This course enhanced my knowledge of the subject matter	1 (1)	0 (0)	1 (1)	6 (8)	72 (90)
The course was relevant to what I might be expected to do (to prevent, prepare for/respond to a trauma) in an emergency department^a^	1 (1)	0(0)	1 (1)	9 (11)	68 (86)
This course provided content that is relevant to my daily job	1 (1)	0 (0)	2 (3)	16 (20)	61 (76)
I would recommend this course to others	1 (1)	0 (0)	0 (0)	4 (5)	75 (94)

a One respondent is missing for this question (N=79).

### KM Level 2: Knowledge and Confidence Matrix Assessments

**Figure 2.**
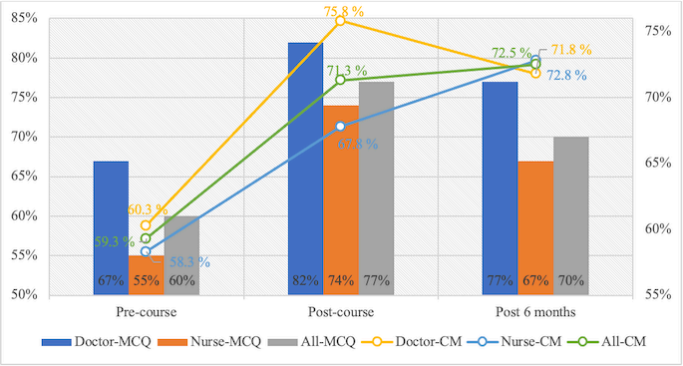
Level-2 assessment of the Kirkpatrick model. The left y-axis shows the percentage of correct multiple-choice questions (MCQs) and the right y-axis shows the percentage of correct responses to the confidence matrix (CM) for the entire cohort and in the nurse and doctor subgroups over time.

There was a significant improvement in correct multiple-choice question responses between the pre- and postcourse assessments in both the doctor and nurse groups, as these scores increased from 67% and 59% to 82% and 74%, respectively (both *P*<.001). Comparing the immediate postcourse assessment to the 6-month follow-up, there was a significant reduction in correct multiple-choice question responses among nurses (74% vs 67%; *P=*.005) but not among doctors (82% vs 77%*; P*=.31). Compared to the precourse scores, the confidence matrix assessment improved significantly immediately following the course in both the doctors and nurses, from 60% to 76% (*P*<.001) and from 58% to 68% (*P*=.02), respectively. Both figures declined after 6 months, although these changes were not statistically significant for doctors (*P*=.07) or nurses (*P=.*51) (Figure 2).

### KM Level 3: Practice Skill Evaluations

#### Scenarios Checklist

In the scenarios section, the average postcourse score in all groups significantly improved (*P*<.001). Six months later, the average scenarios checklist score did not deteriorate (*P*=.99) and this pattern was observed at both hospitals, with *P*=.99 and *P*=.81 for Thanh Hoa and Ninh Binh, respectively (Table 2).

**Table 2.** Scenarios checklist results at the 2 hospitals prior to, immediately following, and 6 months after the course.

Site and assessment		Scenario checklist score, mean (SD)
**All sites**		
	Precourse	5.9 (1.4)
	Post course	9.0 (0.9)
	6-month follow-up	8.5 (0.8)
**Thanh Hoa Hospital**		
	Precourse	6.1 (1.1)
	Post course	9.1 (0.9)
	6-month follow-up	8.4 (0.7)
**Ninh Binh Hospital**		
	Precourse	5.8 (1.6)
	Post course	8.9 (0.8)
	6-month follow-up	8.7 (0.8)

#### Bedside Clinical Checklist

There were 157 possible observed bedside clinical cases precourse and 161 such cases post course at both sites. The bedside clinical scores equally achieved a mean of 5 (SD 1.3) for both sites precourse and increased significantly to 8.4 (SD 0.8) at Ninh Binh and to 8.6 (SD 0.9) at Thanh Hoa, resulting in an overall significant increase of 8.5 (SD 0.8) (*P*<.001) (Table 3).

The responses to all questions showed a significant improvement, with the most significant effects seen for the questions “Is a primary survey/secondary survey undertaken?”, “Was the cervical spine stabilized (manual/collar)?”, and “Was the patient fully exposed and assessed for other injuries?”with changes in percentage post course of 99%, 92%, and 71%, respectively. By contrast, the questions “Was a log roll performed to evaluate the full length of the spine?” and “After any intervention (eg, insertion of an endotracheal tube, treatment of pneumothorax, rapid infusion of fluids) was the ABC (airway, breathing, circulation) reassessed?” showed the lowest rate of correct responses in both pre- and postcourse observed cases, with a change from 1% to 17% and from 10% to 56%, respectively (Table 4).

**Table 3.** Bedside clinical checklist results at the 2 hospitals prior to and immediately following the course.

Site and assessment		Bedside clinical checklist score, mean (SD)
**All sites**		
	Precourse	5 (1.3)
	Post course	8.5 (0.8)
**Ninh Binh Hospital**		
	Precourse	5 (1.3)
	Post course	8.4 (0.8)
**Thanh Hoa Hospital**		
	Precourse	5 (1.3)
	Post course	8.6 (0.9)

**Table 4.** Correct percentages of pre- versus postcourse clinical checklist scores of all observed cases.

Question	Precourse (n=157), n (%)	Post course (n=161), n (%)	Change, %	*P* value
Is a primary survey/secondary survey undertaken?	2 (1.3)	161 (100)	98.7	<.001
Was the cervical spine stabilized (manual/collar)?	11 (7)	160 (99.4)	92.4	<.001
Was oxygen administered/a pulse oximeter probe attached?	111 (70.7)	161 (100)	29.3	<.001
Was the airway assessed? (breathing or not, chest moving or not, obstructed sounds or not)?	131 (83.4)	161 (100)	16.6	<.001
Was the breathing clinically assessed by looking (breath count), feeling (palpation of trachea, percussion of chest), and listening (auscultation)?	123 (78.3)	161 (100)	21.7	<.001
Was the circulation assessed by measurement of heart rate and blood pressure? Was there an assessment of the quality of the pulse, capillary return, and temperature of the peripheries?	140 (89.2)	161 (100)	10.8	<.001
Was blood taken for cross match and hemoglobin/hematocrit analysis? Was an intravenous infusion started?	144 (91.7)	161 (100)	8.3	<.001
Was an AVPU/GCS^a^ neurological assessment of disability done?	89 (56.7)	160 (99.4)	42.7	<.001
Was the patient fully exposed and assessed for other injuries?	3 (1.9)	117 (72.7)	70.8	<.001
Was a log roll performed to evaluate the full length of the spine?	2 (1.3)	30 (18.6)	17.3	<.001
After any intervention (eg, insertion of an endotracheal tube, treatment of pneumothorax, rapid infusion of fluids) was the ABC^b^ reassessed?	15 (9.6)	90 (55.9)	46.3	<.001

a AVPU/GCS: Alert, Voice, Pain, Unresponsive/Glasgow Coma Scale.

b ABC: airway, breathing, circulation.

## Discussion

### Principal Findings

This study demonstrated that the PTC course led to improvements at all 3 levels of the KM. This improvement was maintained for at least 6 months post intervention, except for knowledge in the nurse group. These findings suggest that the knowledge and skills acquired in the PTC course are likely to be translated into clinical practice.

Most of the doctors in the ED who joined the course were junior doctors. This is because, in the Vietnamese medical system, ED work is poorly paid and nonspecialized. In addition, the majority of the nurses and doctors were male. These trends are consistently found in ED staff across the Vietnamese medical system, with most of these staff moving out of the ED into a recognized specialty within a few years [29]. Like many LMICs, ED staffing in the Vietnamese medical system is built on the Franco-German model where staff are not trained as a specialty [30]. Therefore, staff turnover requires frequent redelivery of the PTC course. For this reason, a trauma course such as the PTC course is more suitable for this country. A male predominance among health care staff has also been reported in PTC research of Alwawi (64%), Ologunde (66%), and Nogaro (77%) [31-33]. However, this predominance was not explained in these papers.

### Impact of PTC on Level 1 of the KM: Participants’ Reactions to the Course

Nearly all participants were satisfied with the course. This is in line with the study of Tolppa et al [34], who found that the majority (56/59) of participants agreed or strongly agreed that trauma services are important and 57/59 of participants would recommend the PTC course to their colleagues. In addition, Jawaid et al [35] organized a PTC course with 20 participants, which received a rating of 100% satisfaction, and 100% of the participants also agreed or strongly agreed that their knowledge and skills were enhanced after the course. The authors of these studies argued that having extremely high postcourse ratings was attributed to the course being well-structured/organized as well as having a local champion, along with two other reasons. First, unlike other medical training programs, this course is free of charge. Second, the course is organized locally; therefore, participants were not required to move to other locations, which avoided travel-related logistic issues. Our results are in line with these previous findings and suggest that future courses in limited-resource settings, if organized, should be held locally.

### Impact of the PTC Course on Level 2 of the KM (Knowledge and Confidence Matrix Retention): A Refresh Course on Demand

Our study showed that multiple-choice question scores improved significantly after the course (60% vs 77%; *P*<.001). This finding is similar to those of other studies assessing PTC courses. Amiri et al [36] reported that multiple-choice question scores improved from 63% to 89% for 64 participants comprising physicians and surgeons. Other studies demonstrated significant increases in scores after the course, ranging from 12% to 32% [31,32,35,37-40]. There are 2 multiple-choice questionnaires available in PTC resources (20 and 30 multiple-choice question forms) [25], with comparable results for either form. This reflects the high level of reliability of PTC multiple-choice question tools.

Furthermore, the confidence matrix scores in our study also improved significantly by 17%, which matches the degree of improvement of 20%‐23% reported in previous studies [32,37,39].

However, although multiple-choice question and confidence matrix scores of the doctors and nurses remained unchanged after 6 months, the multiple-choice question scores of nurses declined significantly (74% vs 67%, *P*=.005). This may be explained by the fact that nurses, unlike doctors, are less likely to apply the multiple-choice questions in their routine clinical activities so that there is no reinforcement of these concepts in the workplace after the PTC course. Tolppa et al [34] found that the knowledge gained from a PTC course can be maintained for up to 2 years; this difference from our findings may be related to the fact that Tolppa et al [34] did not report the findings for different subgroups.

### Impact of PTC on Level 3 of the KM: Translation From Acquired Learning to Practice

In simulation situations, the simulation check scores improved significantly from 5.9/10 to 9/10 (*P*<.001). Our result matches that of Jawaid et al [35] on assessing the effectiveness of a PTC course with 20 participants, demonstrating an increase in the median simulation check score from 3.5/20 to 9.5/20 (*P*<.001).

Furthermore, improvement was also seen in clinical application after the course. The bedside clinical checklist score increased significantly from 5/10 to 8.5/10 (*P*<.001). This change demonstrates that the knowledge and skills acquired in the course are effectively converted into clinical practice. In contrast, in a study conducted in El Salvador, Cioè-Peña et al [41] found that despite a significant improvement in the median correct response rate of multiple-choice questions from 74% to 86% after the course, there was no significant change in clinical practice in 194 observed cases for both assessment periods (*P*=.94). This difference could be explained by this study’s different setting along with the different assessment tools and criteria used [41]. Additionally, in the observed cases, we found a significant improvement of all clinical checklist scores (*P*<.001). In particular, the correct response rate for the question “Is a primary survey/secondary survey undertaken?” increased from 1% in the precourse assessment to 100% in the postcourse assessment. This demonstrates that the primary/secondary survey skill, which is a key point of the PTC and other trauma courses (eg, ATLS), was relatively unknown prior to the PTC course, but was greatly improved post course. By contrast, the questions “Was a log roll performed to evaluate the full length of the spine?” and “After any intervention (eg, insertion of an endotracheal tube, treatment of pneumothorax, rapid infusion of fluids) was the ABC reassessed?” demonstrated the least improvement (1% vs 17% and 10% vs 56%, respectively). In the scenarios checklist evaluation, both participant groups demonstrated an understanding of these fundamental aspects. However, in clinical practice, ED staff might still ignore these concepts due to the excess clinical workload. It is recommended that local hospitals adequately support their ED staff to ensure they can provide care to the best of their knowledge and abilities.

Unlike knowledge assessment, it is a long-standing challenge to evaluate clinical activities due to various barriers and obstacles, including time, high cost, availability of skilled clinical supervisors, and other bias/confounding variables such as evaluation and selection bias [42,43]. As we were cognizant of these difficulties, our study was designed to minimize this bias and the potential impacts of confounding factors. However, because the assessment was direct and intermittent, the presence of examiners may have led to a Hawthorne effect, which causes the alteration of behavior by the presence of examinees due to the awareness of being observed [44,45]. It is worth noting that a previous PTC course had been organized in Ninh Binh Hospital in 2008 [17]. Perhaps some residual education and clinical practice effects may have persisted at the time this study was carried out. However, none of the staff who attended the PTC course had been working at the ED prior to when the 2008 course was conducted; thus, a residual educative effect is likely to be negligible or nonexistent.

### Application of the KM and KAP Model in Training

From an educational aspect, among the many factors that affect training program outcomes, the knowledge, attitude, and practice of the trainees are critical components, as they will influence the process of behavioral change, which is the most desired outcome of these courses. In this study, the knowledge, practice, and attitude of health care professionals after the PTC training course were evaluated and showed positive outcomes. When trainees have sufficient and technical knowledge of trauma care, they have positive attitudes and good clinical practice when dealing with trauma patients in the ED. This result is similar to the work of other authors who also applied the KAP approach in their course evaluation [46,47]

Using the KM to evaluate the effectiveness of education/training intervention is not a novel approach [48-50] and it is considered to be more appropriate than other models [51,52]. This study’s results confirmed the effectiveness of PTC training courses at the first 3 levels of the KM, similar to the findings of previous studies [16,34,35]. However, the simple 4-level KM does not help to explain the impact of individual or contextual factors in the evaluation. In the situation of PTC training, contextual factors such as the hospital’s or ED unit’s goals, values, and work environment would impact the application of trained skills on the job of trainees after the courses. In our study, the overloaded situation of the provincial hospitals may be assumed to prevent health care staff from performing the learned procedure to the full capacity when handling trauma patients. In addition, the nature of the tasks of nurses in an ED team may affect their long-term knowledge retention, which could be an expression of the impact of contextual factors on the effectiveness of the training courses. These assumptions warrant deeper investigation in future study, which should consider several organizational factors such as staff turnover, relationships among professionals, and the gender distribution of ED staff. Moreover, in our study, although nearly 100% of the participants were satisfied with the course, which indicates the effectiveness of PTC training courses at the first level of the KM, we do not have evidence to link this positive reaction of the participants to their knowledge transfer and absolute positive postcourse results. A qualitative approach such as in-depth interviews with participants would be useful in detecting the hidden factors that may influence the effectiveness of both the process and outcomes of training, including the organizational aspect of the course, teaching methods, or adequacy of material resources in the courses.

### Limitations

This study has some limitations. To minimize evaluation bias, we informed all examinees of the assessment process. Level 4 of KM is considered a primary endpoint of medical intervention; but, this was not assessed in our study. This will be reported in the subsequent papers by the corresponding author. The study had only a 6 months follow-up and thus lacked a longer evaluation and did not evaluate directly how the actual trauma system changed post intervention. The trauma system includes components such as leadership, professional resources, and financial budget, etc, and therefore may require multiple efforts to be improved [53,54]. Future studies which include these components are required to clarify these issues.

### Conclusions

The PTC course undertaken in 2 provincial hospitals of Vietnam was successful in improving 3 levels of the KM for ED health care staff. This improvement was maintained for at least 6 months after the course. The PTC courses are effective in providing sustained improvement over 3 levels of the KM for LMICs such as Vietnam.

## Supplementary material

10.2196/47127 Multimedia Appendix 1 Level of satisfaction questionnaire.

10.2196/47127 Multimedia Appendix 2 Multiple-choice question test.

10.2196/47127 Multimedia Appendix 3 Confidence matrix.

10.2196/47127 Multimedia Appendix 4 Scenarios.

10.2196/47127 Multimedia Appendix 5 Bedside clinical checklist.

10.2196/47127 Multimedia Appendix 6 Informed consent form.

## Abbreviations

ABC: airway, breathing, circulation
ATLS: Advanced Trauma Life Support
ED: emergency department
KAP: knowledge, attitude, and practice
KM: Kirkpatrick model
LMIC: low- and middle-income country
PTC: Primary Trauma Care
