# Supplementary material for: Using the Kirkpatrick Model to Evaluate the Effect of a Primary Trauma Care Course on Health Care Workers’ Knowledge, Attitude, and Practice in Two Vietnamese Local Hospitals: Prospective Intervention Study
Source: JMIR Med Educ. 2024 Jul 23;10:e47127. doi: 10.2196/47127 (PMC11284612; doi:10.2196/47127)
Supplement: Multimedia Appendix 1 [file mededu-v10-e47127-s001.docx]

Appendix 1

Please circle to the extent in which you agree or disagree with the following statements.

| Question |  | **Strongly Disagree** | **Somewhat Disagree** | **Neither Agree nor Disagree** | **Somewhat Agree** | **Strongly Agree** |
| --- | --- | --- | --- | --- | --- | --- |
| **1** | **I was satisfied with the PTC course overall.** | 1 | 2 | 3 | 4 | 5 |
| **2** | **This course enhanced my knowledge of the subject matter.** | 1 | 2 | 3 | 4 | 5 |
| **3** | **The course was relevant to what I might be expected to do to (prevent, prepare for, or respond to) an emergency department.** | 1 | 2 | 3 | 4 | 5 |
| **4** | **This course provided content that is relevant to my daily job.** | 1 | 2 | 3 | 4 | 5 |
| **5** | **I would recommend this course to others.** | 1 | 2 | 3 | 4 | 5 |
