# Supplementary material for: Using the Kirkpatrick Model to Evaluate the Effect of a Primary Trauma Care Course on Health Care Workers’ Knowledge, Attitude, and Practice in Two Vietnamese Local Hospitals: Prospective Intervention Study
Source: JMIR Med Educ. 2024 Jul 23;10:e47127. doi: 10.2196/47127 (PMC11284612; doi:10.2196/47127)
Supplement: Multimedia Appendix 2 [file mededu-v10-e47127-s002.docx]

| Appendix 2: MCQ test (knowledge assessment) |
| --- |

Q1. Primary Survey should be performed

a. After a secondary survey

b. After rapid ambulance transfer of the patient to a Central Hospital

c. Only at the Central Hospital and not at the District Hospital

d. Within 2 to 5 minutes

e. Within 30 minutes.

Q2 The most common cause of airway obstruction in an unconscious patient is

a. A chest injury

b. A foreign body in the airway

c. The tongue falling to the back of the pharynx

d. A fractured larynx

e. A fractured mandible

Q3. The commonest type of shock in a trauma patient is

a. Cardiogenic shock

b. Hemorrhagic shock

c. Neurogenic shock

d. Septic shock

e. Anaphylactic shock

Q4. Management of a tension pneumothorax detected in the primary survey is

a. Decompression immediately

b. Decompression after chest x-ray confirmation

c. Decompression by chest drain insertion as part of the secondary survey

d. Decompression after anaesthetizing the patient

e. Decompression when tracheal shift develops

Q5. The most reliable method of securing the airway is by using

a. Nasopharyngeal airway

b. Guedel’s airway

c. Laryngeal mask airway

d. Tracheal intubation

e. Esophageal obturator airway

Q6. An early sign of compartment syndrome in a limb is

a. Absent pulse

b. Pain

c. Pale colour

d. Altered sensation.

e. Paralysis

Q7. What is the most likely cause of death following injury to a solid abdominal organ?

a. Haemorrhage

b. Infection

c. Peritonitis

d. Poor function or loss of function of the injured organ

e. Renal failure.

Q8. Which part of the child can be used to estimate the correct tracheal tube size?

a. Ear canal

b. Index finger

c. Nostril

d. Smallest toe

e. Thumb

Q9. In Inhalational injury airway swelling is suggested by

a. Electrical burns to the hands

b. Eye swelling from chemical splash

c. Flash burns to the face

d. Hoarse voice

e. Maxillofacial injury

Q10. An adult male assessed 30 minutes after a road traffic accident has a Glasgow Coma Score of 12/15, a BP of 130/80, a respiratory rate of 13. His right pupil is 5mm diameter with no reaction to light, his left is 3mm and reacting to light. He has a left hemiparesis. The most likely diagnosis is

a. Cervical spinal cord injury at C5

b. Diffuse axonal injury.

c. Cerebral hematoma on the left side

d. Cerebral hematoma on the right side

e. Posterior fossa hematoma.

Q11. During transport, a trauma patient develops severe difficulty breathing, distended neck veins, diminished breath sounds on the right, cyanosis, and deviation of the trachea to the left. Vital signs are blood pressure; 60/40; respirations, 36 per minute; and pulse, 130 per minute. Which of the following is the most appropriate next step?

a. intubates the patient.

b. needles decompress right chest.

c. inserts bilateral chest drains.

d. performs a detailed exam.

e. starts an intravenous line

Q 12. The Secondary survey

a. Will identify haemodynamic instability.

b. Looks for significant injuries that are not an immediate threat to life

c. Includes assessment of AVPU

d. Includes the establishment of intravenous access

e. Should be carried out even if the patient is deteriorating

Q13. Hemothorax

a. Is more common in non-penetrating injury

b. Is associated with increased breath sounds on the affected side

c. Produces tracheal deviation

d. Can result in haemorrhagic shock

e. Always requires thoracotomy

Q14. An adult male with an obvious head injury is comatose. His BP is 170/100, pulse 50 bpm, respiratory rate 24pm. Which is the most likely diagnosis?

a. Hypovolaemia

b. Increased intracranial pressure

c. Drug overdose

d. Pain and anxiety

e. Spinal cord injury.

Q15. A 5 year old child has been struck by a car. She is unconscious with obvious head injuries. Her vital signs on arrival are BP 50/30, pulse 156 bpm, respiratory rate 40 pm. Her weight is estimated as 15 Kg. Initial fluid management is

a. Do not give fluids

b. Give normal saline 50mls bolus

c. Give normal saline 300mls bolus

d. Give O negative blood

e. Wait for cross-matched blood.

Q16. In a patient with greater than 20% by area burns

a. Fluids must be restricted to prevent renal failure

b. The depth of the burn is more significant in resuscitation than the area burned

c. Full thickness burns are more painful than superficial burns

d. Intubation should be avoided with airway burns

e. Crystalloid resuscitation to achieve a urine output of 0.5 to 1.0 mls per Kg per hour is the aim of fluid management.

Q17. In a pregnant trauma patient

a. The patient should be resuscitated in the right lateral position

b. Premature labour is unlikely

c. At 24 weeks the uterus is at the xiphoid cartilage

d. The foetus is less vulnerable as the uterus enlarges

e. Placental separation is an early consequence of blunt abdominal trauma

Q18. Increasing haemodynamic instability is treated by

a. Arrangement of transfer to a major hospital

b. Administering a crystalloid fluid bolus

c. Repeating the secondary survey

d. Performing a neurological examination

e. Administering analgesia using morphine

Q 19. In cervical spine injury

a. Cardiac changes are hypertension and tachycardia

b. Lower limb reflexes are preserved

c. Examination should be carried out in the neutral position

d. Diaphragmatic breathing is preserved in high cervical lesions

e. Assessment of the level of injury does not determine prognosis

Q20. Tracheal intubation must be considered when there is need to

a. Secure an open airway

b. Establish haemodynamic stability

c. Stabilise a cervical fracture

d. Treat a tension pneumothorax

e. Improve peripheral oxygenation in a spontaneously breathing patient.
