# Supplementary material for: Using the Kirkpatrick Model to Evaluate the Effect of a Primary Trauma Care Course on Health Care Workers’ Knowledge, Attitude, and Practice in Two Vietnamese Local Hospitals: Prospective Intervention Study
Source: JMIR Med Educ. 2024 Jul 23;10:e47127. doi: 10.2196/47127 (PMC11284612; doi:10.2196/47127)
Supplement: Multimedia Appendix 3 [file mededu-v10-e47127-s003.docx]

Appendix 3 Confidence matrix

| Please put a ‘X’ in the box that best applies to you 1 = not confident at all 5 = completely confident | | | | | | |
| --- | --- | --- | --- | --- | --- | --- |
|  | How confident do you feel in managing | **1** | **2** | **3** | **4** | **5** |
| A | A 5-year-old child with a fractured pelvis |  |  |  |  |  |
| B | A 30-year-old week pregnant woman with a fractured femur |  |  |  |  |  |
| C | A 25-year-old man with a knife in his abdomen |  |  |  |  |  |
| D | A 60-year-old female with 40 % burns |  |  |  |  |  |
| E | A 50-year-old man unconscious with a fixed dilated pupil |  |  |  |  |  |
| F | A 20-year-old male who is conscious but cannot move his legs |  |  |  |  |  |
| G | A 2-year-old child with major hemorrhage from a traumatic amputation of his leg |  |  |  |  |  |
| H | A 50-year-old man cyanosed from a tension pneumothorax |  |  |  |  |  |

Total point: /40
