# Supplementary material for: Using the Kirkpatrick Model to Evaluate the Effect of a Primary Trauma Care Course on Health Care Workers’ Knowledge, Attitude, and Practice in Two Vietnamese Local Hospitals: Prospective Intervention Study
Source: JMIR Med Educ. 2024 Jul 23;10:e47127. doi: 10.2196/47127 (PMC11284612; doi:10.2196/47127)
Supplement: Multimedia Appendix 4 [file mededu-v10-e47127-s004.docx]

Appendix 4 Scenarios

**SCENARIO 1**

A 35-year-old man has fallen off his motorbike.

On arrival in hospital, he is yelling in pain but is now having difficulty in breathing.

| ASSESSMENT | FURTHER INFORMATION | KEY POINTS |
| --- | --- | --- |
|  |  |  |
| A | Upper airway obstruction signs | 1. Cervical spine |
|  | relieved by simple airway management | 2. Oxygen |
|  | Respiratory rate 28 / min | 3. Airway |
|  |  |  |
| B | Air entry left chest << right | Urgent needle |
|  | Percussion note left >> right | decompression |
|  | Trachea deviated to right | brings some relief |
|  | Tension pneumothorax |  |
|  |  |  |
| C | BP 120/80 | 1. IV line x2 |
|  | Heart rate 100 bpm | 2. Blood sample |
|  |  | 3. Fluid bolus |

During the assessment of the circulation, the airway becomes obstructed.

The patient is now quiet, not yelling out, bag mask ventilation +/- intubation

Diagnosis: Tension pneumothorax

Head injury

|  | **KEY ANSWERS** | **Points** |
| --- | --- | --- |
| **1** | Manual inline neck immobilization |  |
| **2** | Check airway & proceed to airway opening manoeuvre. Give O2 |  |
| **3** | Check breathing – Look , listen and feel – auscultation & percussion |  |
| **4** | Needle decompression 2^nd^ intercostal space L side chest |  |
| **5** | Check BP, pulse, perfusion |  |
| **6** | IV access, take blood sample |  |
| **7** | Reassessment - back to ABC |  |
| **8** | Proceed to mask ventilation - consider endotracheal intubation |  |
| **9** | Neurological examination – AVUP, pupils |  |
| **10** | Call neurosurgeon; arrange CT scan or theatres for burr-hole as required |  |
|  | **TOTAL POINTS** |  |

**SCENARIO 2**

A 45 year-old woman is involved in a head-on collision in a car accident. She was not wearing a seatbelt. She arrives in hospital with dyspnea, difficulty in talking and complaining of right sided chest pain.

| ASSESSMENT | FURTHER INFORMATION | KEY POINTS |
| --- | --- | --- |
|  |  |  |
| A | Obstructed airway  (gurgling, snoring) | Cervical spine care  Jaw thrust |
|  | Respiratory rate 30x | Suction |
|  |  | Oxygen |
|  |  |  |
| B | No air entry on right side | Needle decompression |
|  | Trachea deviated to left | +/- chest tubes |
|  | Tension pneumothorax | results in |
|  |  | RR 16x |
|  |  | Able to talk again |
|  |  |  |
| C | BP 110/70 | Fluid bolus x2 |
|  | HR 110 bpm | Blood test |
|  |  | IV line x2 |
|  |  | Results in |
|  |  | BP 120/80 HR 90 |

Diagnosis: Fractured ribs + left tension pneumothorax

Cervical spine injury

|  | **KEY ANSWERS** | **Points** |
| --- | --- | --- |
| **1** | Manual inline neck immobilization |  |
| **2** | Check airway & proceed to airway opening manoeuvre |  |
| **3** | Clear airway with suctioning |  |
| **4** | Apply high flow oxygen |  |
| **5** | Assess breathing with auscultation and percussion |  |
| **6** | Identify and treat tension pneumothorax with needle thoracocentesis |  |
| **7** | Assess circulation – HR, BP, CRT |  |
| **8** | Insert 2 x IV cannulae & collect blood for x match |  |
| **9** | Fluid bolus x 2 |  |
| **10** | Secondary Survey |  |
|  | **TOTAL POINTS** |  |

**SCENARIO 3**

A 25-year-old man falls off motorbike at high speed, not wearing a helmet.

Arrives in hospital, gurgling, noisy shallow breathing, unconscious. Left thigh is swollen.

| ASSESSMENT | FURTHER INFORMATION | KEY POINTS |
| --- | --- | --- |
|  |  |  |
| A | Stops breathing during assessment | Cervical spine care |
|  |  | Airway support |
|  |  | Bag mask Ventilation |
|  |  | or intubate |
|  |  | Oxygen |
|  |  |  |
| B | Chest is clear | No chest injury |
|  | Air entry equal |  |
|  | Percussion equal |  |
|  |  |  |
| C | HR 110 | IV line 2x |
|  | BP 120/80 | Blood test |
|  |  | Fluid bolus |
|  |  |  |
| D | Pupils initially fixed + dilated | Consult Neuro- surgeon |
|  | Back to normal with Oxygen |  |
|  |  |  |
| E | Closed femur fracture loss | Replace blood |
|  |  | Immobilize/splint |

Secondary survey

Diagnosis: Head injury (diffuse axonal injury)

Fractured femur

|  | **KEY ANSWERS** | **Points** |
| --- | --- | --- |
| **1** | In line neck stabilization |  |
| **2** | Size for cervical collar |  |
| **3** | Clear airway |  |
| **4** | Support ventilation |  |
| **5** | Intubate patient |  |
| **6** | Check for chest injuries |  |
| **7** | IV line X 2 |  |
| **8** | Check AVPU |  |
| **9** | Check for dilated pupil |  |
| **10** | Immobilise leg |  |
|  | **TOTAL POINTS** |  |

**SCENARIO 4**

A 19 year-old man has been stabbed in the abdomen. On arriving in hospital, he is clutching his abdomen and complaining of severe abdominal pain.

| ASSESSMENT | FURTHER INFORMATION | KEY POINTS |
| --- | --- | --- |
|  |  |  |
| A | Airway clear | Oxygen |
|  | Cervical spine OK |  |
|  |  |  |
| B | Air entry diminished on the right | Chest drain |
|  | Percussion note dull on the right | blood >> |
|  | (Hemothorax) |  |
|  |  |  |
| C | BP 80/40 | IV line 2x |
|  | HR 120 | Blood test |
|  | Pale and sweaty | Fluid bolus x2 |
|  |  |  |
|  | No response to fluid | 1. Transfusion |
|  | still hypotensive | 2. Call surgeon |
|  |  | 3. Prepare operating theatre |

Diagnosis: Penetrating chest and abdominal trauma with life threatening haemorrhage

|  | **KEY ANSWERS** | **Points** |
| --- | --- | --- |
| **1** | Check airway |  |
| **2** | Give Oxygen |  |
| **3** | Chest auscultation and percussion |  |
| **4** | Insertion of chest drain – location : 5^th^ intercostal, anterior to midaxillary line |  |
| **5** | Check HR and BP |  |
| **6** | IV access & samples for Hb and cross-match |  |
| **7** | IV fluid bolus |  |
| **8** | Start blood transfusion |  |
| **9** | Call for surgeon and preparation of theatre |  |
| **10** | Abdominal examination |  |
|  | **TOTAL POINTS** |  |

**SCENARIO 5**

A 26 year-old woman is shot in the neck. She arrives in hospital, conscious but with stridor and respiratory distress.

| ASSESSMENT | FURTHER INFORMATION | KEY POINTS |
| --- | --- | --- |
|  |  |  |
| A | Stridor | Oxygen |
|  | Difficulty in talking | Simple airway management |
|  | Hoarse voice |  |
|  |  |  |
| B | Air entry equal but soft |  |
|  | Chest clear |  |
|  |  |  |
| C | BP 120/80 |  |
|  | HR 110 |  |

During assessment of her circulation you notice the neck has become more swollen. There is increasing stridor and she now is unable to talk.

Surgical consultation for tracheostomy

Consider cricothyroidotomy

Do not attempt intubation

Diagnosis: Gunshot wound to larynx

|  | **KEY ANSWERS** | **Points** |
| --- | --- | --- |
| **1** | Manual inline immobilization of neck |  |
| **2** | Introduce yourself and talk to patient |  |
| **3** | Notice hoarse voice, air hunger, laboured respiratory effort |  |
| **4** | Give Oxygen by mask |  |
| **5** | Look, listen & feel: cyanosis, bil chest air entry, breathe sound. Feel trachea |  |
| **6** | Check pulse and BP |  |
| **7** | Access IV X 2. Take samples for: FBC, glucose, U&E, Cross-match. Start IV fluids |  |
| **8** | Cover gunshot wound with gauze. Notice swelling , subcutaneous emphysema |  |
| **9** | Perform cricothyroidotomy |  |
| **10** | Arrange theatre for surgical cricothyroidotomy / tracheostomy & surgical exploration |  |
|  | **TOTAL POINTS** |  |

**SCENARIO 6**

A 60 year-old woman is involved in a high speed car accident. She was wearing a seatbelt. On arrival in hospital she is groaning in pain. BP 90/70, HR 130, RR 28. She had cold hands and feet.

| ASSESSMENT | FURTHER INFORMATION | | KEY POINTS |
| --- | --- | --- | --- |
| A | Soft, obstructed breathing  spine care | | Cervical management |
|  | Spits out Guedel airway if put in  Basic airway | | Oxygen |
| B | Chest clear | |  |
| C | BP 80/60, HR 140 | | IV line 2x |
|  | Fluid bolus 2 L: BP 110/70 HR 110 | | Blood for tests |
|  | After 1 L --> BP 120/80 HR 80 | |  |
|  | After 2 L - ->Patient responds well to fluid bolus. | |  |
| D | Now consciousness becomes normal |  | |

Secondary survey: tender pelvis with crepitus on palpation

Diagnosis: Fractured pelvis

|  | **KEY ANSWERS** | **Points** |
| --- | --- | --- |
| **1** | Manual neck inline immobilization |  |
| **2** | Give Oxygen |  |
| **3** | Basic airway management – chin lift / jaw thrust |  |
| **4** | Check breathing – chest auscultation |  |
| **5** | Check HR and BP |  |
| **6** | Access IV and take samples for FBC and cross-match. Start IV fluids |  |
| **7** | Recheck HR and BP – further IV fluids |  |
| **8** | Conscious level - AVUP |  |
| **9** | Secondary survey – fractured pelvis – pelvic sling |  |
| **10** | Analgesia |  |
|  | **TOTAL POINTS** |  |

**SCENARIO 7**

A 15 year-old boy climbing a tree fell to the ground, landing on his head.

According to his family, he was initially conscious and complained of neck pain. Over the next 30 minutes he became unconscious. On arrival in hospital he had a right side seizure.

| ASSESSMENT | FURTHER INFORMATION | KEY POINTS |
| --- | --- | --- |
| A | Obstructed breathing (snoring, some distress) | Cervical spine care  Oxygen |
|  |  | Simple airway management but may need intubation |
| B | Shallow breaths | Patient needs ventilatory assistance with a bag and mask |
| C | BP 130/90 HR 100 | IV access 2x  Blood tests |
| D | Left pupil fixed + dilated  Flexing to pain only | Call neurosurgeon  Prepare burr-hole |

Diagnosis: Left extradural hematoma

|  | **KEY ANSWERS** | **Points** |
| --- | --- | --- |
| **1** | Manual in line cervical spine immobilization |  |
| **2** | Clear airway & Open airway – chin lift |  |
| **3** | Give Oxygen |  |
| **4** | Look, listen and feel breathing - auscultation |  |
| **5** | Ventilation assistance. Consider intubation |  |
| **6** | Check circulation - BP, pulse and perfusion |  |
| **7** | IV access and take blood samples for FBC |  |
| **8** | AVUP and neurological examination |  |
| **9** | Call neurosurgeon - Arrange theatre for hematoma evacuation |  |
| **10** | Proceed to secondary survey |  |
|  | **TOTAL POINTS** |  |

**SCENARIO 8**

A 45 year-old factory worker is crushed by a container truck at work. He is brought into hospital with severe breathing difficulties.

| ASSESSMENT | FURTHER INFORMATION | KEY POINTS |
| --- | --- | --- |
| A | Respiratory rate 40  Shallow breathing  Cyanosed | Cervical spine care  Simple airway management  Oxygen |
| B  intubation | Bilateral wheeze and crepitation  Air entry << on right side  Paradoxical movement on right anterior  chest (flail chest) | Needs for flail chest  Analgesia  Percussion note dull on right side  Drain blood from the right chest |
| C | BP 100/60 HR 140  Good response to fluid | IV access 2x  Blood tests  Fluid bolus x2 |

Diagnosis: Crush injury with right sided flail chest and haemothorax

|  | **KEY ANSWERS** | **Points** |
| --- | --- | --- |
| **1** | Check response from head end |  |
| **2** | In line cervical spine immobilization |  |
| **3** | Look, listen and feel for breathing |  |
| **4** | Clear airway, Guedel’s airway |  |
| **5** | Give Oxygen |  |
| **6** | Right side chest tube |  |
| **7** | 2 I/V line access by 18 G cannula |  |
| **8** | I/V Fluids |  |
| **9** | Blood sample for cross match and FBC, Urea, sugar. |  |
| **10** | Analgesia |  |
|  | **TOTAL POINTS** |  |

**SCENARIO 9**

A 25 year old woman has been assaulted with a large heavy stick. On arrival in hospital she has extensive facial injuries, stridor and respiratory distress. She is cyanosed and is making groaning sounds.

ASSESSMENT FURTHER INFORMATION KEY POINTS

A Basic airway management does not Cervical spine care

relieve the airway obstruction Oxygen

Bag mask ventilation is difficult Suction, jaw thrust, Chin lift

Bag mask ventilation

Must intubate

B Chest: air entry normal

Breathing sounds OK

C BP 130/90 HR 110 IV line

Blood tests

Fluid bolus

D Pupils equal and normal response

to light

Open eyes to voice (remembers)

Localizes to pain

Secondary survey: swollen right humerus

Diagnosis: Facial fractures,

Moderate blood loss,

Fractured humerus

|  | **KEY ANSWERS** | **Points** |
| --- | --- | --- |
| **1** | Apply Oxygen |  |
| **2** | Basic airway management – Chin lift / Jaw thrust |  |
| **3** | Suction Airway |  |
| **4** | Bag mask ventilation |  |
| **5** | Requires intubation / Consider possible need for cricothyroidotomy |  |
| **6** | Requires sedation / anaesthesia / analgesia |  |
| **7** | C Spine care during intubation attempt – Manual in line stabilization |  |
| **8** | Assess breathing and circulation |  |
| **9** | Moderate hypovolaemic shock / IV access and fluid bolus |  |
| **10** | Proceed to secondary survey |  |
|  | **TOTAL POINTS** |  |

**SCENARIO 10**

A 70 year old man has been burnt in a house fire. It has taken 4 hours for the patient to reach hospital. On arrival, he is still dressed with a burnt area covering his chest and abdomen. He is groaning in pain and appears to be in respiratory distress. He is confused.

ASSESSMENT FURTHER INFORMATION KEY POINTS

A No facial or airway burns Oxygen

on inspection Airway managment

Respiratory rate 30 SpO2 88% on air

B Wheeze on auscultation

Air entry normal R = L

C BP 90/70 HR 130 IV access 2x

Cold periphery Blood tests

Fluid bolus

- good response

D Alert, oriented after oxygen

Secondary survey 30-40% burns Burns care Resuscitation

Tetanus Prophylaxis

Supportive treatment

Diagnosis: Burns 30-40% with inhalation injury to the lungs.

|  | **KEY ANSWERS** | **Points** |
| --- | --- | --- |
| **1** | Check for face, neck and upper airway burns |  |
| **2** | Check breathing pattern – Respiratory rate, auscultation, O_2_ Sats |  |
| **3** | Give Oxygen by mask |  |
| **4** | Check circulation – BP, pulse, perfusion |  |
| **5** | IV access, and take blood samples for FBC, E&E, glucose, cross-match |  |
| **6** | Give IV fluid boluses – check response |  |
| **7** | Secondary survey – exclude other injuries |  |
| **8** | Calculate % burn area |  |
| **9** | Calculate fluids requirement according to burn area. Attention to urine output |  |
| **10** | Attention to: burn care, tetanus cover, analgesia, supportive treatment |  |
|  | **TOTAL POINTS** |  |

**SCENARIO 11**

A 25 year-old man has been injured in an explosion and fire at a factory. He is on his way to hospital with facial burns and a chest injury.

ASSESSMENT FURTHER INFORMATION KEY POINTS

A Hoarse voice Cervical spine care

Burns around and in his mouth Oxygen

Carbonaceous sputum prepares for intubation

No stridor, RR 30

B Air entry: reduced on right Chest drain

Percussion dulls on right Hemothorax Tender to palpation right chest

Rib fractures

C BP 90/60 IV access 2x

HR 120 Blood for tests

IV Fluid

During assessment of circulation, patient develops increasing stridor. If he has not been intubated, return to A.

A Unable to talk, stridor Intubate if not already done

Respiratory distress

Diagnosis: Upper airway and facial burns with possible cervical spine injury

Right rib fractures and haemothorax

|  | **KEY ANSWERS** | **Points** |
| --- | --- | --- |
| **1** | Manual immobilization cervical spine |  |
| **2** | Check and prepare for intubation |  |
| **3** | Apply high flow oxygen |  |
| **4** | Assess breathing: auscultation and percussion |  |
| **5** | Identify and treat haemothorax with chest drain |  |
| **6** | Assess circulation: HR, BP, CRT |  |
| **7** | Insert 2 x IV cannulae; collect blood for X Match |  |
| **8** | Administer fluid bolus |  |
| **9** | Consider pain relief |  |
| **10** | Calculate fluids for next 24 hours |  |
|  | **TOTAL POINTS** |  |

**SCENARIO 12**

An 8 year-old boy is riding a bicycle and is hit by a car. He is brought to the hospital and is complaining of abdominal pain.

ASSESSMENT FURTHER INFORMATION KEY POINTS

A Airway clear, Respiratory rate 24 Cervical spine care.

Oxygen

B Air entry R = L

Percussion note R = L

Expansion R = L

Chest sounds normal

C BP 70/40 HR 140 IV line 2x

Periphery cold Blood for tests

Capillary return 4 seconds Fluid bolus 20ml/kg

- BP 80/60 HR 120

After next 20ml/kg

- 100/70 HR 90

Develops obstructed breathing and becomes drowsy while you are assessing C

A Obstruction relieved by airway, bag and mask

B Satisfactory

C Stable

D Fixed dilated pupil right side. Not responding to pain

Diagnosis: Blood loss from an unknown site. Right side extradural hematoma

|  | **KEY ANSWERS** | **Points** |
| --- | --- | --- |
| **1** | Cervical spine care |  |
| **2** | Give Oxygen |  |
| **3** | Notice cold peripheries |  |
| **4** | Check capillary refill |  |
| **5** | Give 20 ml/kg IV fluid bolus |  |
| **6** | Give 2^nd^ IV fluid bolus |  |
| **7** | Relieve airway obstruction |  |
| **8** | Look at pupils |  |
| **9** | Keep patient warm |  |
| **10** | Take bloods for cross-match |  |
|  | **TOTAL POINTS** |  |

**SCENARIO 13**

A 26 year old, 30 week pregnant woman is involved in a motor vehicle accident. On arrival in hospital, she is complaining of abdominal pain.

ASSESSMENT FURTHER INFORMATION KEY POINTS

A Respiratory rate 20 Oxygen

Able to talk Cervical spine care

Complaining of dyspnea Left lateral position

B Air entry normal

Percussion normal

C BP 90/60 HR 140 IV line 2x

Blood for tests

Fluid bolus 2x

Blood pressure remains low

if not resuscitated in left lateral

position.

Good response to fluid

D Normal

Secondary survey Tender abdomen

Fundal height at xiphisternum

Fetal heart sounds are not heard

Diagnosis: Uterine trauma with placental abruption and separation

|  | **KEY ANSWERS** | **Points** |
| --- | --- | --- |
| **1** | Check airway and give Oxygen |  |
| **2** | Check cervical spine |  |
| **3** | Deviation of uterus to the left (left lateral position) |  |
| **4** | Check breathing: auscultation , percussion |  |
| **5** | Check circulation: HR and BP |  |
| **6** | IV access and take blood samples for Hb and cross-match |  |
| **7** | Give IV fluid bolus |  |
| **8** | Check response to fluid bolus: HR and BP |  |
| **9** | Abdominal examination |  |
| **10** | Call obstetrician |  |
|  | **TOTAL POINTS** |  |

**SCENARIO 14**

A 45 year-old male prisoner is stabbed in the back in a fight. He is unable to move his legs and is having problems breathing. He is complaining of pain in the right chest.

ASSESSMENT FURTHER INFORMATION KEY POINTS

A Able to talk with difficulty Oxygen

Airway is clear

B Air entry R < L Decompress right side

Percussion note R > L Tension pneumothorax

Trachea deviated to left

C BP 90/60 HR 120 IV access 2x

Blood for tests

Fluid bolus x2

BP becomes 90/60 HR 100

D Alert

Secondary survey Reflexes absent in lower limbs

Sensory level loss up to T8

Diagnosis: Spinal injury of T7-T8

Tension pneumothorax

|  | **KEY ANSWERS** | **Points** |
| --- | --- | --- |
| **1** | Manual inline neck immobilization |  |
| **2** | Introduce yourself & talk to patient. Notice clear airway but laboured breathing |  |
| **3** | Give Oxygen by mask |  |
| **4** | Look, listen & feel. Cyanosis, ↓ air entry R side chest . Feel trachea. Percussion. Auscultation |  |
| **5** | Decompress R side chest – wide bore needle in 2^nd^ intercostal space, mid clavicular |  |
| **6** | Check pulse and BP |  |
| **7** | Access IV X 2. Take samples for FBC, glucose, U&E and cross-match. Start Ringers |  |
| **8** | Arrange formal chest tube thoracostomy |  |
| **9** | Chack disability – AVUP and neurological examination |  |
| **10** | Secondary survey – log roll. Wound examination, spine, sensory loss below T8 |  |
|  | **TOTAL POINTS** |  |

**SCENARIO 15**

A 32-year-old woman has fallen from a cliff on a remote island. It has taken 4 days for her to reach hospital. She has an obvious compound fracture of her left femur and a swollen left calf. The leg smells. She appears very confused.

ASSESSMENT FURTHER INFORMATION KEY POINTS

A Airway clear. Respiratory rate 30 Oxygen

Cervical spine care

B Chest normal

C BP 100/40 HR 120 IV access

Bounding pulse Blood tests

Temperature 39 Celsius Fluid bolus x2

Good response to fluid

D Confused

Secondary survey Pulseless, cold left foot Fasciotomy

Antibiotics

Diagnosis: Septic shock from compound fracture

Compartment syndrome left calf

|  | **KEY ANSWERS** | **Points** |
| --- | --- | --- |
| **1** | Check airway and breathing |  |
| **2** | Give Oxygen |  |
| **3** | Check pulse and BP |  |
| **4** | Access IV. Take samples for FBC, U&E, glucose and cross-match |  |
| **5** | Start IV fluids |  |
| **6** | Check conscious level - AVUP |  |
| **7** | Respond to fluids – HR< BP and conscious level |  |
| **8** | Secondary survey – notice fractured L femur, and cold – pulseless foot |  |
| **9** | Arranged theatre for fasciotomies |  |
| **10** | Start antibiotics and analgesia |  |
|  | **TOTAL POINTS** |  |

**SCENARIO 16**

A 4 year old girl has been run over by a car. She is brought into the Emergency department straight away. She is not breathing.

ASSESSMENT FURTHER INFORMATION KEY POINTS

A No breath sounds Cervical spine care

No chest movement Bag mask ventilation

Unable to ventilate Intubation

B After intubation, notice no chest Urgent needle

movement on right side Decompression

Percussion note resonant on right Chest drain for hemopneumothorax

Trachea deviated to left

C BP 60/50 HR 130 Intra-osseus needle

Capillary return slow Fluid bolus 2x

Unable to put in IV line

on 2 attempts

Good response BP 90/60 HR 100

D Now open eyes and biting ETT

Diagnosis: Head injury

Right haemopneumothorax

|  | **KEY ANSWERS** | **Points** |
| --- | --- | --- |
| **1** | Check response, airway, breathing |  |
| **2** | In line cervical spine immobilization |  |
| **3** | Bag mask ventilation |  |
| **4** | Endo tracheal intubation |  |
| **5** | Needle decompression R second intercostal space |  |
| **6** | Formal R Chest tube insertion |  |
| **7** | Intra osseous needle insertion |  |
| **8** | Collect blood sample for cross match, FBC, Sugar, urea |  |
| **9** | Fluid bolus |  |
| **10** | Secondary survey |  |
|  | **TOTAL POINTS** |  |

**SCENARIO 17**

A 40 year old man is the driver in a car accident. He was ejected from the car and was found 20 metres away. On arrival in hospital

ASSESSMENT FURTHER INFORMATION KEY POINTS

A Stridor and respiratory distress Cervical spine care

Loose teeth and blood on oral suction Oxygen

Simple airway management

Bag mask ventilation

Airway management is not effective

B Ventilation with bag mask not effective Attempt intubation

A Attempted intubation failed 2x

Now patient is cyanosed Cricothyrotomy

Plan: Follow with tracheostomy

Continue Primary Survey (ABCD)

|  | **KEY ANSWERS** | **Points** |
| --- | --- | --- |
| **1** | Basic airway management – Chin lift / Jaw thrust |  |
| **2** | Suction Airway |  |
| **3** | Requires intubation |  |
| **4** | Bag mask ventilation |  |
| **5** | C Spine care during intubation attempt – Manual in line stabilization |  |
| **6** | Requires cricothyroidotomy |  |
| **7** | Assess breathing and circulation |  |
| **8** | High risk of other internal injuries with ejection |  |
| **9** | IV access |  |
| **10** | Trauma X rays if possible – C Spine, Chest, Pelvis |  |
|  | **TOTAL POINTS** |  |
