# Supplementary material for: Using the Kirkpatrick Model to Evaluate the Effect of a Primary Trauma Care Course on Health Care Workers’ Knowledge, Attitude, and Practice in Two Vietnamese Local Hospitals: Prospective Intervention Study
Source: JMIR Med Educ. 2024 Jul 23;10:e47127. doi: 10.2196/47127 (PMC11284612; doi:10.2196/47127)
Supplement: Multimedia Appendix 5 [file mededu-v10-e47127-s005.docx]

Appendix 5: Bedside clinical checklist

| N^0^ | Question | Y/N |
| --- | --- | --- |
| A | Is a Primary Survey/ Secondary Survey undertaken? |  |
| 1 | Was the Cervical Spine stabilized (manual/collar)? |  |
| 2 | Was oxygen administered/ a pulse oximeter probe attached? |  |
| 3 | Was the Airway assessed? (Breathing or not, chest moving or not, obstructed sounds or not?) |  |
| 4 | Was the Breathing clinically assessed by looking (breath count) feeling (palpation of trachea, percussion of chest) and listening (auscultation)? |  |
| 5 | Was the Circulation assessed by measurement of heart rate and blood pressure? Was there an assessment the quality of the pulse, capillary return, and temperature of the peripheries? |  |
| 6 | Was blood taken for cross match and hemoglobin/hematocrit analysis? Was an IV infusion started? |  |
| 7 | Was an AVPU/GCS neurological assessment of Disability done? |  |
| 8 | Was the patient fully Exposed and assessed for other injuries? |  |
| 9 | Was a log roll performed to evaluate the full length of the spine? |  |
| 10 | After any intervention (e.g. insertion of an endotracheal tube, treatment of pneumothorax, rapid infusion of fluids) was the ABC reassessed? |  |

Total point: /10
