# Supplementary material for: Using the Kirkpatrick Model to Evaluate the Effect of a Primary Trauma Care Course on Health Care Workers’ Knowledge, Attitude, and Practice in Two Vietnamese Local Hospitals: Prospective Intervention Study
Source: JMIR Med Educ. 2024 Jul 23;10:e47127. doi: 10.2196/47127 (PMC11284612; doi:10.2196/47127)
Supplement: Multimedia Appendix 6 [file mededu-v10-e47127-s006.docx]

Appendix 6: Informed consent

**PARTICIPANT INFORMATION AND CONSENT SHEET**

(For staff)

**General information**

**PISTACHIO** (The Primary Trauma Care Course Impact on Healthcare worker expertise and Patient Outcome in Vietnam) is a project which aims to evaluate the impact of trauma training on the healthcare system and patient outcomes. The results of this study will inform us on trauma training which has been used for more than 20 years internationally, without an assessment of how well it works.

**Research team**

Principal Investigator: Prof. Mark Nelson ([Mark.Nelson@utas.edu.au](mailto:Mark.Nelson@utas.edu.au)), Menzies Institute for Medical research, University of Tasmania

Local supervisor: Prof. Nguyen Huu Tu ([nguyenhuutu@hmu.edu.vn](mailto:nguyenhuutu@hmu.edu.vn)), Hanoi Medical University

PhD student: Nguyen Ba Tuan, M.D ([Batuan.nguyen@utas.edu.au](mailto:Batuan.nguyen@utas.edu.au)), Menzies Institute for Medical research, University of Tasmania

**Why have I been invited to participate?**

You have been invited because you are staffs of emergency department/hospital. We will investigate how trauma management changes after our training.

**What will I be asked to do?**

You will be asked to do the MCQ test, self-interviewed for level of confident while managing trauma patient, practice in scenarios and real patient in the ED.

**How many people will take part in?**

All ED staff of your hospital, including you.

**Are there any possible risks from participation?**

As mentioned above, your answer for our question will be collected. The risk of leaking private information will be eliminated as it will be coded and stored securely in google drive’s account with pass code. All identifying information will be removed.

**Will my information be confidential?**

All your information will be considered as confidential.

**What will happen to the information when this study is over?**

The answer paper will be converted to an electronic version, which will then be kept at Hanoi Medical University (HMU) store. It, then, will be deleted after 10 years as per HMU regulations. The electronic version will be sent to the University of Tasmania via “one drive” and can be stored longer for future research activity.

**How will the results of the study be published?**

The data will be published as a paper, report, or other types without any identifiable information**.**

**Will I be informed the result of this study?**

As a participant you have right to ask for it by contact with the researcher via phone or email below.

**How can I agree to be involved?**

If you would like to participate, please fill and sign the consent form.

**What if I have the question about this project?**

If you have any question, please to contact with

1. Dr. Nguyen Ba Tuan (PhD student) at [nguyenbatuan@utas.edu.vn](mailto:nguyenbatuan@utas.edu.vn) or via phone: +84936568824
2. Dr. Phung Lam Toi (research assistant) at [toiphunglam@gmai.com](mailto:toiphunglam@gmai.com) or via phone: +84 963625068

Thank you for your time!

**By signing this form, I agree that:**

1. I am voluntarily taking part in this project. I understand that I do not have to take part, and I can stop at any time.

2. The result of the test, practice, checklist points may be used as described above.

3. I have read the Information sheet

4. I do not expect to receive any benefit or payment for my participation.

5. I can request a copy of my result if needed and may make edits I feel necessary to ensure the effectiveness of any agreement made about confidentiality

6. I have been able to ask any questions I might have, and I understand that I am free to contact the researcher with any questions I may have in the future.

| Date | ……../………/202… |
| --- | --- |
| Participants Signature |  |
| Printed name |  |
| Researchers Signature |  |
